# The Early Diagnosis of Gastric Cancer

**Published:** 1916-06

**Authors:** John R. Williams

**Affiliations:** Rochester, N. Y.


### BUFFALO MEDICAL JOURNAL

Yearly Volume 71 JUNE, 1916 No. 11

### ORIGINAL ARTICLES

**The right is reserved to decline papers not dealing with practical medical and surgical subjects, and such as might offend or fail to interest readers. Contributors are solely responsible for opinions, methods of expression and revision of proof.**

### The Early Diagnosis of Gastric Cancer.

### Suggestions to the General Practitioner.

By JOHN R. WILLIAMS, M. D., Rochester, N. Y.

The absolute clinical diagnosis of early gastric cancer is impossible in the present stage of our medical knowledge. The diagnosis of early gastric cancer when made must be largely conjectural and based on probabilities. What was formerly considered an early stage of cancer is now known to be the moderately advanced or hopeless period. By early stage of gastric cancer we mean that phase of the disease where a few cancer cells have become implanted in an area of irritation in the gastric mucosa. Such an area of irritation including the cancer cells may not be larger than a fifty cent piece or even much smaller. A cancer area so small as this may fail to produce symptoms. The most striking symptom produced by cancer is a toxemia, which expresses itself as cachexia, loss of weight, and loss of appetite. Pain as a symptom of cancer of the stomach depends largely on the position of the growth. The same may be said of nausea and vomiting. In the early stage of gastric cancer none of these phenomena may be present.

Although the actual cause of cancer is not known, we are in possession of some information concerning its etiology. Practically all clinical surgeons believe that gastric cancer in the majority of instances develops on the site of an old ulcer. In the Mayo clinic, approximately seventy per cent of gastric cancers are believed to have been pre-ulcerous. Moynihan, an opponent of this teaching at first reluctant to believe it, now estimates that 72 per cent of his cases were preulcerous. Mayo Robson reports that 59 per cent of his cases were pre-ulcerous. These surgical clinicians agree that the proportion of cases of cancer developing on the site of ulcer depends directly upon the training of the clinician and the thoroughness of his methods of investigation. If these statements be accepted as a working guide in the diagnosis of early cancer it is obvious that the physician must give his attention to the study of gastric ulcer. The way therefore to cure gastric cancer is to prevent if possible or cure gastric ulcer.

Until Rosenow in 1914 proved that gastro-intestinal ulcer could be caused by streptococcic infection starting from foci in the mouth, there was no adequate explanation of the cause of gastro-intestinal ulcer. How frequently mouth infection is the causal agent in producing gastric ulcer is problematical, but that the conditions are commonly associated has been frequently observed. During the past thirty months I have seen 13 cases of gastro-intestinal ulcer which have come to operation. Of these 12 had severe dento-alveolar infection. The following case is illustrative:

Case 704, male iron worker, aged 50, presented himself on November 3, 1913, for examination, complaining of pain and distress in the epigastrium and irregular movements of the bowels. A clinical examination was made at the time, from which it was concluded that he had chronic ulcer in the upper gastro-intestinal tract. It was observed at the time that his teeth were badly decayed, in many cases nothing but rotten stumps remaining. The gums were swollen and edematous; the tongue swollen and teeth-marked; breath foul; cervical glands moderately enlarged.

On December 23, 1913, patient had severe hemorrhage from the stomach, vomiting blood until he was almost lifeless. The patient was immediately removed to the hospital. On December 27th he was operated upon by Dr. Thomas Jameson and a duodenal ulcer about the size of a 25 cent piece was found one and one-half inches from the pylorus. Just before operation a blood culture was made, also a culture from a pus pocket between the gum and one of the infected teeth. At operation a swollen lymph gland, lying in close proximity to the ulcer, was removed with aseptic precautions and placed in a sterile tube. Later this gland was crushed with a sterile rod and the juice pressed therefrom was used to inoculate agar slants. A streptococcus was isolated from this gland juice. which resembled morphologically and culturally an organism recovered from the blood and the infected teeth. The organism was slow growing and died out before its identity could be established.

If the absolute clinical diagnosis of early cancer is at the present time impossible, it is proper to ask, what is the basis of a conjectural diagnosis? When a middle-aged individual presents himself complaining of loss of appetite, loss of weight, and of indisposition, the physician should assume, until he can prove otherwise, that he is dealing with a case of malignancy, and he should make a most exhaustive examination of such a patient, calling to his aid the experience of men specially trained in the various fields of scientific endeavor.

In the examination for suspected gastric cancer, the most important element is the history of the case. Such a history should be comprehensive and in its relation to the digestive functions should so particularize as to fix definitely every important event in the gastro-intestinal history of the patient. A detailed record of all preceding attacks of gastro-intestinal disturbance should be secured. Careful inquiry should be made into such illnesses as ptomaine poisoning, dysentery, stomach ache, heartburn, and gas on the stomach, for many patients and physicians mistake these disorders for gastro-intestinal ulcer.

The specific history of pain should receive careful attention. Its relation to the meal is important. Pain in the mid-epigastrium occurring three or four hours after meals, especially if it radiates to the right, is likely to be duodenal ulcer. Pain occurring an hour or two after meals in the mid-epigastrium and perhaps radiating to the left, is more likely to be gastric ulcer. Pain of this character is commonly relieved by food or alkali in the stomach, because both of these neutralize the acid which causes the irritation of the ulcer. Pain from an ulcer on the posterior surface of the stomach, especially where there are adhesions to the pancreas, commonly causes pain in the shoulder. It is impossible to make a diagnosis of ulcer or cancer on the basis of pain alone, because pain in the stomach resulting from spasm of the pylorus may be caused by many other conditions, as appendicitis, gall bladder disease, kidney disease, and gastric neuroses. The proper value to be attached to the pain symptom can however be ascertained when these other disease phenomena are excluded.

The reason for loss of appetite should be carefully considered. Tuberculosis and many other bacterial or parasitic infections and primary anemia may cause loss of appetite.

These can only be excluded by careful special examinations. The same may be said of loss of weight.

Vomiting is a sign of lesser importance. Its cause is largely mechanical, and it bears no relation to the stage of or the severity of either ulcer or cancer. An ulcer may develop near the pylorus in which cancer cells may or may not become implanted. The area of infiltration through interference with the circulation may lead to edema of the adjacent stomach mucosa, the resulting suffering may close the pylorus so as to form either a partial or total obstruction. Such cases have been reported. The character and quantity of the vomitus has significance. If the patient regurgitates merely a small amount of clear, colorless, acid fluid, it means that there is no obstruction in the pylorus, and that hemorrhages, if occurring, are very minute. If he regurgitates a large quantity of old food material, light brown or chocolate color, it means that the patient has stasis or dilation of the stomach, probably obstruction together with a hemorrhage.

The absence of a history of gastro-intestinal disorder does not preclude the possibility of cancer. In 1913 I had a patient referred to me, who had just returned from a well known clinic where he had sought advice with reference to certain bladder symptoms caused by prostatic disease. He had no gastro-intestinal symptoms. In my routine examination I discovered evidence of gastric cancer and so reported. The patient was then referred to Dr. Roswell Park, of Buffalo, who urged exploratory incision in view of my report. This was done and a hopeless condition of malignancy was found in the stomach. It is important, therefore, to remember that the history does not always reveal evidence of gastric cancer.

Next in importance to the history taking, in the examination of a case of this type, is the physical examination. This should include a careful examination of the mouth and head sinuses, and if there is the least suspicion of concealed infection, the services of a specialist should be enlisted. Suspicion should be directed against crowned teeth, bridges, receded gums and dead teeth. The tonsils should be carefully explored, and the physician should not consider his examination in these complete until he has made the patient gag so as to project the tonsils into full view. A negative history of tonsilitis does not preclude severe low grade tonsilar infection. A cryptic tonsil covered with mucus or filled with cheesy plugs, particularly if submerged and lying beneath the pillars of the fauces, may be regarded as harboring deleterious infection.

One of the best premonitory signs of mouth infection is a swollen, teeth-marked tongue with palpable cervical glands. I have had an opportunity of examining several hundred persons with reference to mouth infections, and I have never yet seen a patient with a swollen, teeth-marked tongue and palpable cervical glands where head infection could not be demonstrated.

The importance of mouth infection cannot be over-estimated in the investigation of gastro-intestinal disease. Recently, I was asked to examine a male, 62 years of age, who was suffering from loss of appetite, loss of weight, marked cachexia, nausea and vomiting. The patient presented a very satisfactory picture of malignancy, and his physician, with gastro-intestinal cancer in mind, was contemplating operative treatment. In my examination I discovered that the man had thirteen badly infected and rotten stumps in his mouth. I gave the opinion that if the man's illness were due to carcinoma, his condition was hopeless and beyond surgical aid, but that if it were due to mouth infection, removal of the infected teeth would undoubtedly promote recovery. The patient was then referred to a dentist. Since the removal of the teeth the patient has made a rapid and complete recovery. I have seen eight such cases during the past two years.

A routine examination of the lungs, heart and arteries should then be made and disease therein excluded. In the examination of the abdomen I have worked with practically all of the examining tables on the market, and have come to the conclusion that the very best one is a dining-room chair. When the stripped patient is seated on such a chair and is made to lean over so as to almost rest the chest on his knees, the physician, standing behind the patient, can explore the relaxed abdomen in a most surprising manner. He can reach up under the liver and into the deep recesses of the pelvis by this method in a way which is impossible with a patient lying on a flat table, in a hot bath, or even in a rocking-chair, as has been suggested by some clinicians. In palpating the abdomen the examiner should always begin with the flat of his hand. To commence such a study by punching the finger tips into the abdominal wall is to excite muscular reflexes, which are very likely to defeat the purpose of the investigator. When the patient is examined by this method it is easy to distinguish a diseased appendix, a sore kidney, or a swollen and tender gall bladder from a stomach lesion. An enlarged spleen or a neoplasm in the gastro-intestinal tract can be felt in this way where by other palpation methods these could not be detected.

The laboratory investigations of early gastric cancer afford little positive evidence. In the early days of gastro-intestinal studies it was believed possible to recognize and differentiate the majority of cases of gastric cancer. We now know that the evidence which was formerly considered positive pertained to the advanced or hopeless stage of the disease. Nevertheless, it may be said with truth that the chemical and biological study of the gastro-intestinal tract in advanced cancer affords more convincing evidence than does any other line of study. Its value in the early stages, however, is only corroborative. I have had experience in the examination of upwards of three thousand gastro-intestinal cases, many of which proved to be cancer. My experience is quite similar to that of Smithies', whose observations are probably the most accurate and conclusive to be found in the literature, because he has had an unusual opportunity to follow up and confirm his findings. They are essentially as follows:

If the growth interferes with the motor power of the stomach, the amount of stomach contents recovered will be greater than normal in proportion to the degree of obstruction. Free hydro-chloric acid is usually lower than normal and may be absent. Occasionally it may be excessive. Lactic acid is rarely present in the early stage of cancer. Occult blood is present in about half of the early cases. The Oppler-Boas organism is not commonly seen in the early stage unless there is interference with the emptying of the stomach. Then its presence is quite striking. An early state of ulcer may present such evidence as retention, rancid food, low or absent hydrochloric acid, the presence of lactic acid and occult blood. The glyclytryptophan test, because of ease of error in the test, is of very doubtful value. The Abderhalden biological test for cancer is so uncertain in its present stage of development as to be worthless.

The next step in the study of a gastro-intestinal case is the X-ray examination. This is perhaps the most valuable single laboratory aid. Since it belongs in the field of special study and its employment is beyond the technical capacity of the majority of general practitioners, it is not here considered.

Summary and conclusions:

1. A positive clinical diagnosis of the early stage of gastric cancer is practically impossible at the present time.

2. There is no single sign or group of pathognomonic signs pointing indubitably to early gastric cancer.

3. A very large per cent of gastric cancers develop on the site of old ulcers. It is in this pre-cancerous or ulcerative stage that gastric malignancy should be recognized.

4. Loss of appetite and loss of weight, nausea and vomiting in a middle-aged person should always excite the suspicion of the physician that he is dealing with gastro-intestinal malignancy. These are the signs, however, of moderately advanced lesions.

5. A conjectural or probable diagnosis may frequently be made after an exhaustive examination of the patient, of which a careful life history of the individual is of paramount importance. History taking should never be delegated to inexperienced or untrained assistants. A careful physical examination, while not affording positive evidence of early gastric cancer, is valuable in excluding many other conditions giving rise to similar disease phenomena. Laboratory investigations are valuable in so far as they afford corroborative evidence and evidence excluding other possible causes.

6. Gastric cancer is of such a serious nature and its course so rapid that palliative medical treatment is a most dangerous expedient, and should never be resorted to to the exclusion of the scientific investigations that have been described in this paper. If a patient under suspicion does not make a rapid complete recovery even in the face of all negative laboratory evidence, his abdomen should be opened.

7. Gaylord has pointed out that immunity to invasion of cancer is infinitely greater in the early stage of the disease than it is in its later phases. Everyone is agreed that the only hope afforded the cancer victim is the early surgical eradication of his disease.

8. These facts impose a tremendous responsibility upon the clinician, and he who denies his patient the assistance afforded by the present-day sciences shirks his responsibility, and invites misery and death to his patient.

---

**Exacerbation of Latent Syphilis and Gonorrhoea in the Course of Wounds by Fire Arms.** Henri Toussaint, Soc. Chir., Nov. 3, 1915, cites three cases of syphilis showing renewed virulence of the treponema in symbiosis with common pyogens. He does not believe that exacerbation of venereal diseases will prove as frequent as that of malaria, alcoholism (delirium tremens?) and tuberculosis but suggests that the surgeon should not intervene till the system has been sterilized by medical action. Lumbar puncture, the Wassermann reaction, leucoplasia, black tongue and hemeralopic pigmentary retinitis are mentioned as diagnostic points. (Note: The author does not, of course, imply neglect of emergent surgical procedures nor undue delay in diagnostic procedures. His language is partly figurative, referring to the clearing of a region by one force before another goes to the attack, and is difficult of exact translation without implying a crude conception of infectious processes).

---

**Acidosis in Children.** Edward Clay Mitchell of Memphis, Memphis Med. Monthly, Dec. 1915. Children of gouty, rheumatic and alcoholic parents are predisposed, and there seems to be a family tendency. Carbohydrate starvation is the usually accepted cause. He reports seven cases, three in one family, with 3 deaths. Treatment consists in lavage, soda by mouth and hypodermatically, withdrawal of animal foods and administration of glucose solutions by rectum.

